# Identification of *Leuconostoc* species based on novel marker genes identified using real-time PCR *via* computational pangenome analysis

**DOI:** 10.3389/fmicb.2022.1014872

**Published:** 2022-09-23

**Authors:** Eiseul Kim, Seung-Min Yang, Ik-Seon Kim, So-Yun Lee, Hae-Yeong Kim

**Affiliations:** Department of Food Science and Biotechnology, Institute of Life Sciences and Resources, Kyung Hee University, Yongin, South Korea

**Keywords:** computational pangenome analysis, identification, real-time PCR, species-specific genes, *Leuconostoc*

## Abstract

*Leuconostoc* species are important microorganisms in food fermentation but also cause food spoilage. Although these species are commercially important, their taxonomy is still based on inaccurate identification methods. Here, we used computational pangenome analysis to develop a real-time PCR-based method for identifying and differentiating the 12 major *Leuconostoc* species found in food. Analysis of pan and core-genome phylogenies showed clustering of strains into 12 distinct groups according to the species. Pangenome analysis of 130 *Leuconostoc* genomes from these 12 species enabled the identification of each species-specific gene. *In silico* testing of the species-specific genes against 143 publicly available *Leuconostoc* and 100 other lactic acid bacterial genomes showed that all the assays had 100% inclusivity/exclusivity. We also verified the specificity for each primer pair targeting each specific gene using 23 target and 124 non-target strains and found high specificity (100%). The sensitivity of the real-time PCR method was 10^2^ colony forming units (CFUs)/ml in pure culture and spiked food samples. All standard curves showed good linear correlations, with an *R*^2^ value of ≥0.996, suggesting that screened targets have good specificity and strong anti-interference ability from food sample matrices and non-target strains. The real-time PCR method can be potentially used to determine the taxonomic status and identify the *Leuconostoc* species in foods.

## Introduction

The genus *Leuconostoc* belongs to the Lactobacillaceae family, also known as lactic acid bacteria. They inhabit several food sources, such as vegetables, silage, fruits, meat, fish, and dairy products (de Paula et al., 2015). *Leuconostoc* species can metabolize numerous sugars, alcohols, and carbohydrates and are used as a flavor starter in many fermented products (Guglielmotti et al., 2022). Specifically, they improve the physicochemical properties of fermented foods by producing organic acids, volatile compounds, and CO_2_, which contribute to the texture and flavor of dairy products (cheese, butter, and cream) (de Paula et al., 2015). Therefore, *Leuconostoc* species are important food microorganisms that positively influence food fermentation. However, certain species have detrimental effects and cause food spoilage (Hemme and Foucaud-Scheunemann, 2004). For example, some *Leu*. *mesenteroides* strains were considered opportunistic pathogens causing pulmonary infection and peritonitis and some *Leu*. *gasicomitatum* strains were identified as spoilage organism for broiler meat strips (Susiluoto et al., 2003; Menegueti et al., 2018). *Leuconostoc* species have traditionally been used as a probiotic candidates due to their ability to survive in the unfavorable conditions of the gastrointestinal tract (Björkroth et al., 2014). Although some *Leuconostoc* species are considered safe for use in the food industry and are known as “generally recognized as safe (GRAS)” organisms, they have been linked with diseases in immunocompromised patients (Kumar et al., 2022).

After undergoing several re-arrangements, the taxonomy of *Leuconostoc* currently includes 17 species,^1^ of which the following 12 were primarily isolated from food matrices: *Leuconostoc* (*Leu.*). *carnosum*, *Leu*. *citreum*, *Leu*. *fallax*, *Leu*. *gasicomitatum*, *Leu*. *gelidum*, *Leu*. *holzapfelii*, *Leu*. *inhae*, *Leu*. *kimchii*, *Leu*. *lactis*, *Leu*. *mesenteroides*, *Leu*. *pseudomesenteroides*, and *Leu*. *suionicum* (Hemme and Foucaud-Scheunemann, 2004; Padilla-Frausto et al., 2015). This genus has undergone several reclassifications. While some species that were originally classified under the genus *Leuconostoc* were reclassified as *Oenococcus, Fructobacillus,* and *Weissella*, others have been considered heterotypic synonyms (Björkroth et al., 2014; Bello et al., 2022). Most recently, the previously reported *Leu*. *mesenteroides* strain was reclassified as *Leu*. *suionicum* based on whole-genome-based sequence information (Kaushal and Singh, 2020). Another recent study suggested reclassifying *Leu*. *gelidum* subsp. *gasicomitatum* as *Leu*. *gasicomitatum* species (Wu and Gu, 2021). *Leuconostoc* species are often found in similar habitats as *Lactococcus* and *Lactobacillus* related species and were considered intermediates between *Lactobacillus* and *Streptococcus* (Kumar et al., 2022). Although *Leuconostoc* species are widely used along with *Lactobacillus*, its taxonomy is relatively less explored than *Lactobacillus*.

The selecting target genes and developing specific primer pairs are crucial factors for achieving accurate real-time PCR results (Martínez-Romero et al., 2018). Several previously described specific genes for *Leuconostoc* (16S rRNA gene, 23S rRNA gene, and 16S–23S intergenic region, *hsp60*, and *rpoB* genes) were considered while designing the specific primers (Kaur et al., 2017; Ricciardi et al., 2020; Guglielmotti et al., 2022). Several authors have found ribosomal genes (16S rRNA and 23S rRNA) problematic as they do not exhibit sufficient variability to allow differentiation between closely related species (Ricciardi et al., 2020; Guglielmotti et al., 2022). In the case of the genus *Leuconostoc*, a high degree of similarity (98.5 ~ 99.7%) was reported between the gene sequences for 16S rRNA (Wu and Gu, 2021). These authors also reported that some species, such as *Leu*. *gelidum*, *Leu*. *gasicomitatum* and *Leu*. *inhae* exhibit sequence similarities of 99% or higher (99.1–99.7%) between their 16S rRNA gene sequences. These facts highlight the need to find alternative specific genes to identify *Leuconostoc* species accurately.

Researchers have identified novel DNA markers to replace current, poorly specific markers using whole-genome sequencing-based methods. Whole-genome sequencing is widely used to determine the taxonomic position of microorganisms (Kumar et al., 2022). Recent comparative genomic studies have confirmed that the average nucleotide identity (ANI) and digital DNA–DNA hybridization (dDDH) might suffice to classify *Leuconostoc* species at the species or subspecies level (Wu and Gu, 2021; Bello et al., 2022). Also, more robust approaches (e.g., pan-and core-genome analysis) were used to account for strain diversity and classify closely related species or subspecies (Kim et al., 2021, 2022b,c). Although this method can provide the resolution necessary to identify bacterial genera or species within food samples, simpler methods such as real-time PCR provide higher resolution at lower cost and shorter testing time (Wang et al., 2022). Real-time PCR methods are advantageous for identification and differentiation compared with whole-genome sequencing as they are rapid, specific, and sensitive and do not require post-PCR processing. The food industry-accepted methods must be easy to use, affordable, and accurately provide species-level resolution.

Here, we developed an easy-to-use and accurate real-time PCR method based on novel marker genes obtained from computational pangenome analysis that can be used to identify the 12 *Leuconostoc* species that predominantly inhabit food matrices and are essential for food fermentation.

## Materials and methods

### Genome sequences

Whole-genomic sequences of 130 strains, including 13 *Leu*. *carnosum*, 10 *Leu*. *citreum*, 3 *Leu*. *fallax*, 14 *Leu*. *gasicomitatum*, 14 *Leu*. *gelidum*, 2 *Leu*. *holzapfelii*, 5 *Leu*. *inhae*, 2 *Leu*. *kimchii*, 12 *Leu*. *lactis*, 34 *Leu*. *mesenteroides*, 14 *Leu*. *pseudomesenteroides*, and 7 *Leu*. *suionicum* were retrieved from the National Center for Biotechnology Information (NCBI) database (last accessed on April 11, 2022) (Supplementary Table S1). The selection criteria for involving only 130 strain genome sequence data are as follows: the genome assembled at the complete level was preferentially used, and the species without complete genomes was used in order of scaffold and contig.

### Phylogenetic analysis

To ensure proper classification of all genomes, the pangenome and phylogenetic analysis were performed using the bacterial pangenome analysis (BPGA) tool version 1.3.0 (Chaudhari et al., 2016). Protein-coding gene sequences from each genome were used as input for the analysis. The identity cut-off was used as the default value (0.5) for the similarity calculation (Lim et al., 2021). MUSCLE (Multiple Sequence Comparison by Log-Expectation), a tool built into BPGA, was used for the phylogeny analysis. Core-genomic and pangenomic phylogenies were constructed based on linked core gene alignments and pan matrix (binary gene presence/absence matrix) and visualized using the interactive tree of life program version 6 (Letunic and Bork, 2021). The ANI was calculated using JSpeciesWS version 3.9.5 (Richter et al., 2016).

### Screening species-specific genes

The species-specific genes were identified using a pangenome analysis pipeline. Briefly, assembled genomes were annotated using Prokka version 1.14.5 (Seemann, 2014), and these annotated assemblies, obtained in GFF3 format, were applied to calculate the pangenome analysis using Roary version 3.11.2 (Page et al., 2015). The pangenome was analyzed for each genome using a BLASTP identity cut-off of 95%. The presence/absence profiles of genes were converted into a 0/1 matrix using a script available with Roary. The matrix was employed to screen *Leuconostoc* species-specific genes according to the following criteria: 100% presence in target genomes and absence in non-target genomes. The identified specific genes were further selected from the whole-genome shotgun contigs databases using the nucleotide basic local alignment search tool (BLAST) version 2.13.0 with default parameters (word_size = 28, expect threshold = 0.05, and max matches in a query range = 0). The primers targeting each species-specific gene were designed using Primer Designer program (Scientific and Education Software, Durham, NC, United States).

### Bacterial strains

We used 147 reference strains that were purchased from the Korean Collection for Type Cultures (KCTC, Daejeon, South Korea), the Korean Culture Center of Microorganisms (KCCM, Seoul, South Korea), the Korean Agricultural Culture Collection (KACC, Jeonju, South Korea), and the Korean Collection for Kimchi Microorganisms (KCKM, Gwangju, South Korea) (Supplementary Table S2).

The reference strains were cultured in MRS broth at 37°C for 48 h. The cells were collected by centrifugation at 13,000 × *g* for 5 min, and DNA was extracted using the DNeasy Blood & Tissue kit (Qiagen, Hilden, Germany) according to the manufacturer’s instructions. The concentration of the extracted DNA was estimated using the Maestro Nano-spectrophotometer (Maestrogen, Las Vegas, NV, United States). The genomic DNA was stored at −20°C until real-time PCR analysis.

### Evaluation of real-time PCR assay

A real-time PCR assay was performed in CFX96 Deep Well Real-Time System (Bio-Rad, Hercules, CA, United States) using a mixture consisting of 10 μl 2× A-Star Master Mix (BioFACT, Daejaon, South Korea), 1 μl of each primer pair (500 nM), 50 ng of template DNA, and distilled water up to a final volume of 20 μl. The real-time PCR conditions were initial denaturation at 95°C for 5 min, followed by 35 cycles at 95°C for 5 s and 60°C for 30 s. Melting curves were constructed by continuously increasing the temperature from 65°C to 95°C in 0.5°C increments, at 5 s per step. The real-time PCR assay was performed in triplicate.

### Specificity and sensitivity

We evaluated the specificity and amplification efficiency of the developed real-time PCR method, which helped us determine the lowest detectable DNA concentration.

The inclusivity/exclusivity of primer pairs was first assessed by *in silico* PCR^2^ analysis with genome sequence data of 133 target and 110 non-target strains obtained from GenBank. The specificity of the primer pairs was also evaluated using pure bacterial DNA. Genomic DNA was extracted from 147 lactic acid bacterial strains and used as a template.

Standard curves were established using serially diluted target bacterial strains ranging from 10^8^ to 10^1^ CFU/ml (Gómez-Rojo et al., 2015). For genomic DNA extraction, 1 ml of each dilution was taken and plated on lactobacilli MRS agar (Difco, Detroit, MI, United States) to determine the correlation between CFU/ml and the Ct value (Bustin et al., 2009). The amplification efficiency was calculated based on the formula: Efficiency = 10^1/slope^−1 (Bustin et al., 2009).

### Detection of *Leuconostoc* in spiked foods

Pork, lettuce, and pasteurized milk were purchased from the local markets in Korea. All samples were previously tested for the presence of 12 *Leuconostoc* species by real-time PCR. When not all *Leuconostoc* species were detected, these three samples were used for spiking with *Leuconostoc* species to prepare the contaminated samples. The cultured bacterial strains were diluted to concentrations from 10^8^ to 10^1^ CFU/ml with phosphate buffered saline (PBS). The cocktail of 12 *Leuconostoc* reference strains at concentration of 10^8^ to 10^1^ CFU/ml each was prepared. The bacterial cell number in the dilutions was confirmed by plate counting method. For pork meat and lettuce samples, 25 g of each sample was placed in stomacher bag and the cocktail was inoculated. After standing for 10 min, samples were homogenized with 225 ml PBS. Non-inoculated food samples were used as a negative control through the same procedure. The DNA extraction was conducted according to the method described in section “*Bacterial strains*” without additional incubation procedures and then analyzed using real-time PCR under the conditions described in section “*Evaluation of real-time PCR assay*.”

## Results and discussion

### Phylogenetic analysis

Bacterial pangenome analysis was used to construct phylogenetic tree based on pan and core-genome to confirm that the taxonomic labels of genomes used for analysis were correct. Roary was used to identify species-specific genes based on gene presence/absence matrix.

**Figure 1:**
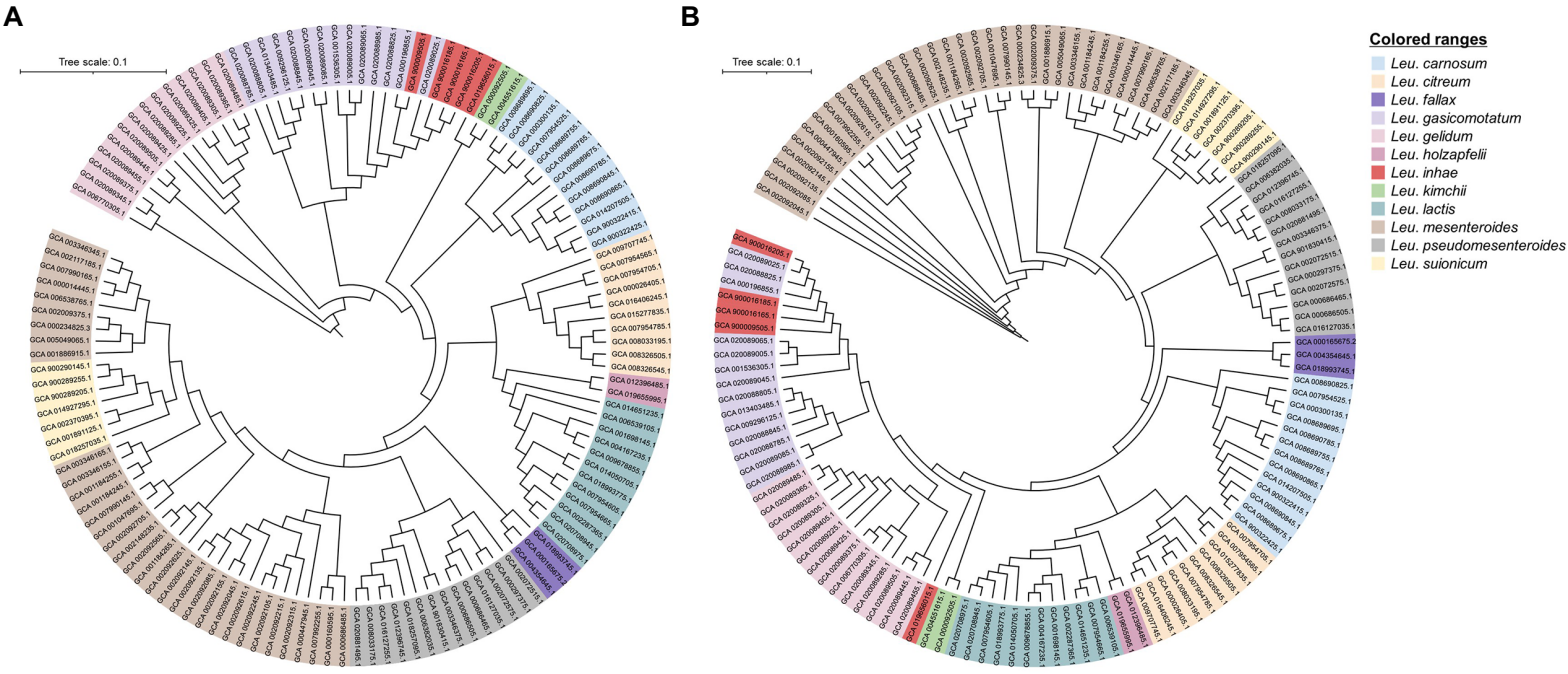
Phylogenetic relationship between 130 *Leuconostoc* strains. **(A)** Pangenome phylogenetic tree based on binary panmatrix (gene presence/absence (1/0) matrix). **(B)** Core-genome phylogenetic tree based on concatenated core gene alignment. Trees were visualized using Interactive Tree of Life software.

A total of 130 *Leuconostoc* genomes were obtained from the GenBank database, and the genomes were clustered using a phylogenetic tree based on pan and core genomes (Figure 1). Both methods subdivided the samples into 12 large clusters according to their species name Four genomes did not cluster with the same species, suggesting the species name was incorrect. These were *Leu*. *inhae* strains, which were *Leu*. *gasicomitatum*. The cluster containing *Leu*. *gelidum* group species was divided into *Leu*. *gelidum*, *Leu*. *gasicomitatum*, *Leu*. *inhae*, and *Leu*. *kimchii* strains. However, the *Leu*. *inhae* PB1a (GCA_900016185.1), KSL4-2 (GCA_900016165.1), PL111 (GCA_900016205.1), and C120c (GCA_900009505.1) strains clustered with *Leu*. *gasicomitatum* strains. In the phylogenetic tree based on pangenome, four *Leu*. *inhae* strains (PB1a, KSL4-2, PL111, and C120c) were clustered with *Leu*. *gasicomitatum* strains, whereas one *Leu*. *inhae* strain (DSM 15101) was clustered with *Leu*. *kimchii*. In the phylogenetic tree based on core-genome, four *Leu*. *inhae* strains (PB1a, KSL4-2, PL111, and C120c) were clustered with *Leu*. *gasicomitatum* strains, which was consistent with the pangenome tree, and *Leu*. *inhae* DSM 15101 existed independently between *Leu*. *kimchii* and *Leu*. *gelidum*. Therefore, a comparison of core and pan-based trees showed differences in order within species cluster but no differences in species assignment, which is consistent with previous study (Akwani et al., 2022).

**Figure 2:**
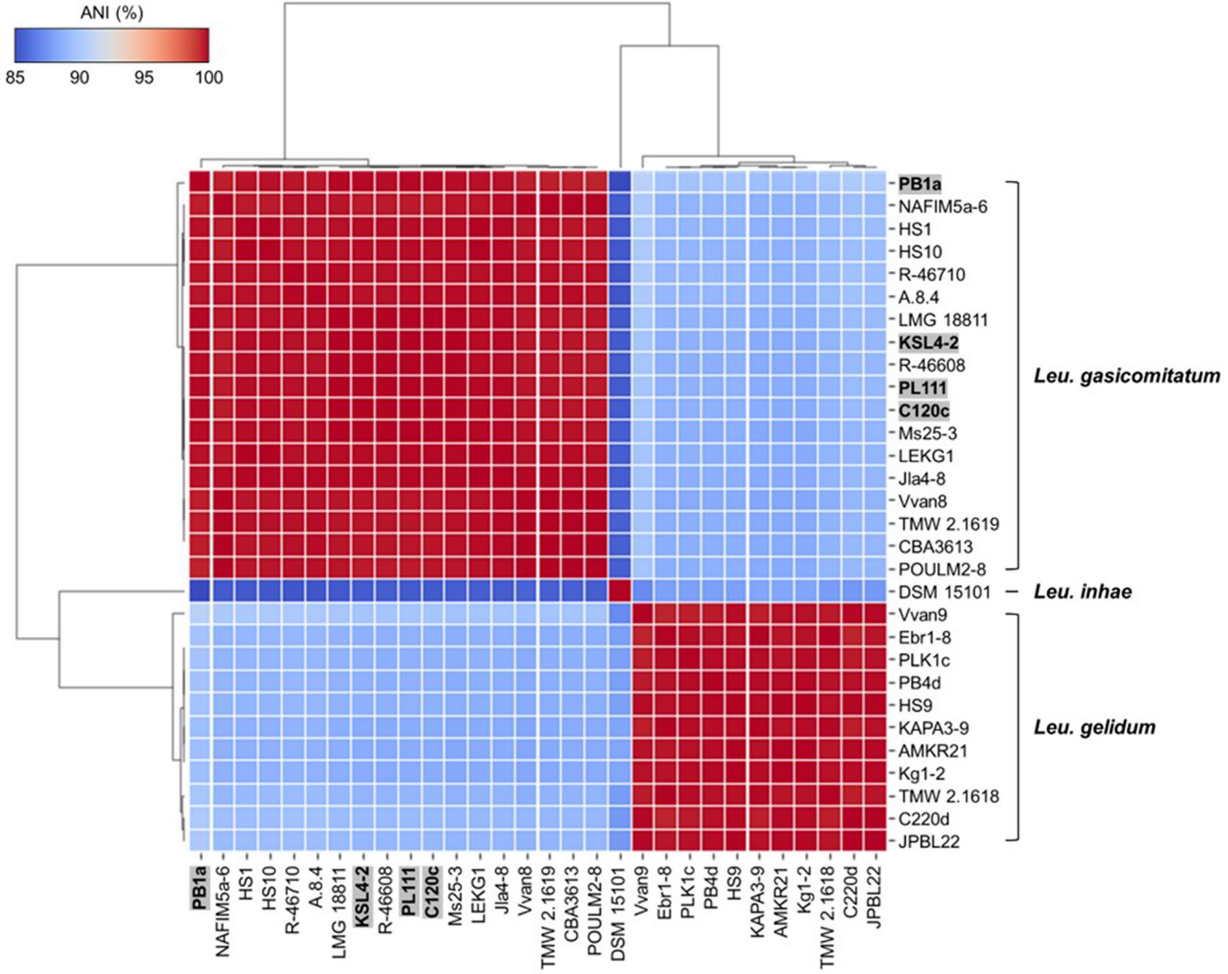
Heatmap of average nucleotide identity (ANI) values among *Leu*. *gelidum*, *Leu*. *gasicomitatum*, and *Leu*. *inhae* strains. Misclassified genomes are highlighted in grey box with bold letters. Strain names and accession numbers are listed in Supplementary Table S1.

Average nucleotide identity analysis showed more than 95% identity among the same species, whereas less than 95% identity between different species (Supplementary Table S3). However, *Leu*. *inhae* PB1a, KSL4-2, PL111, and C120c strains had 86.94–87.1% identity with *Leu*. *inhae* type strain (DSM 15101^T^) while 99.04–99.62% identity with *Leu*. *gasicomitatum* type strain (LMG 18811^T^) (Figure 2). Also, *Leu*. *inhae* PB1a, KSL4-2, PL111, and C120c strains showed more similarity with other *Leu*. *gasicomitatum* genomes (97.72 to 99.96% identities) than *Leu*. *inhae* genome (86.94 to 87.1% identities).

Consistently, previous studies reported that incorrectly assigned taxonomic labels for bacterial species are prevalent with reported (Ghosh et al., 2019; Yang et al., 2021; Akwani et al., 2022). For example, *Lacticaseibacillus paracasei* was misclassified as *Lacticaseibacillus casei* and *Enterococcus lactis* as *Enterococcus faecium*, all of which are closely related species (Ghosh et al., 2019; Kim et al., 2022a). Critically, inaccurate genomic information might impede the development of methods distinguishing *Leuconostoc* species, so the information for the strains (*Leu*. *inhae* PB1a, KSL4-2, PL111, and C120c) should be corrected in the GenBank database to prevent further misidentification.

### Screening species-specific genes

Recently, PCR methods targeting specific genes were developed from comparative genomics to accurately identify closely related species within genera. Pangenome analysis helps in finding more DNA markers for identifying closely related species (Belloso Daza et al., 2021). Highly specific genes identified *via* pangenome analysis are useful alternative genetic markers for differentiating closely related species. Thus, molecular assays targeting genetic markers achieve higher resolution within closely related species. The previous researchers have successfully identified for species, subspecies, or serovar-specific marker genes using the presence/absence matrix with a script built into Roary (Bannantine et al., 2021; Shang et al., 2021; Kim et al., 2022c). Here we selected novel species-specific genes that facilitate more accurate identification than the 16S rRNA gene and housekeeping genes using pangenome analysis.

A total of 130 genomes were used for the pangenome analysis and were clustered using gene presence in the accessory genome. The pangenome was screened for *Leuconostoc* species-specific genes using a gene presence/absence matrix, with species-specific genes defined as being present in 100% of the target species and absence in other species. The number of core genes in each species varied from 781 to 1,939 (Supplementary Table S4). We identified 783 species-specific genes, of which 42, 64, 305, 3, 3, 77, 106, 76, 18, 2, 68, and 19 were specific to *Leu*. *carnosum*, *Leu*. *citreum*, *Leu*. *fallax*, *Leu*. *gasicomitatum*, *Leu*. *gelidum*, *Leu*. *holzapfelii*, *Leu*. *inhae*, *Leu*. *kimchii*, *Leu*. *lactis*, *Leu*. *mesenteroides*, *Leu*. *pseudomesenteroides*, and *Leu*. *Suionicum*, respectively. These were further tested against 84,780,734 sequences using the BLAST nr/nt database and nine genomes representing phylogenetically related *Leuconostoc* species to target species (i.e., *Leu*. *falkenbergense*, *Leu*. *rapi*, *Leu*. *litchii*, *Leu*. *miyukkimchii*, and *Leu*. *palmae*). This reduced the number marker genes to 7 *Leu*. *carnosum*, 8 *Leu*. *citreum*, 34 *Leu*. *fallax*, 1 *Leu*. *gasicomitatum*, 1 *Leu*. *gelidum*, 8 *Leu*. *holzapfelii*, 40 *Leu*. *inhae*, 16 *Leu*. *kimchii*, 3 *Leu*. *lactis*, 1 *Leu*. *mesenteroides*, 10 *Leu*. *pseudomesenteroides*, and 1 *Leu*. *suionicum*-specific genes. Among these, we selected genes specific to each species based on their GC content and length (Table 1).

**Table 1:** Information of *Leuconostoc* species-specific genes obtained from pangenome analysis.

Target species	Target gene	Accession number
*Leu. carnosum*	Accessory secretory protein Asp2	AFT80940.1
*Leu. citreum*	Glycerophosphoryl diester phosphodiesterase	ACA83530.1
*Leu. fallax*	Hypothetical protein	TDG67205.1
*Leu. gasicomitatum*	Transcriptional regulator, TetR family	CUR64472.1
*Leu. gelidum*	Restriction endonuclease	QDJ29634.1
*Leu. holzapfelii*	Accessory sec system glycosyltransferase GtfA	NKZ17642.1
*Leu. inhae*	3,4-dihydroxy-2-butanone-4-phosphate synthase	WP_220734157.1
*Leu. kimchii*	Acyl-CoA thioesterase 1, truncated	ADG39653.1
*Leu. lactis*	DUF2316 family protein	RYS85616.1
*Leu. mesenteroides*	Peptidase	AET29880.1
*Leu. pseudomesenteroides*	3-dehydroquinate dehydratase I	KDA48106.1
*Leu. suionicum*	Hypothetical protein	API72908.1

### *In silico* specificity

Primer pairs designed from species-specific genes are shown in Table 2. Twelve specific genes were confirmed using *in silico* PCR with the 143 *Leuconostoc* genomes and 100 other lactic acid bacteria genomes. For each species, we selected primer pairs representing specific genes of each species that showed 100% inclusivity and exclusivity in the *in silico* PCR (Supplementary Table S5). The amplicon sizes ranged from 100 to 211 bp. These primers were further tested using real-time PCR.

**Table 2:** Twelve *Leuconostoc* species-specific primer pairs used in this study.

Target species	Primer name	Primer sequence (5′-3′)	Size (bp)
*Leu. carnosum*	CA_F	GAC CGT CAG GCA CCG CTT AT	135
	CA_R	GGC GCC ACC TTG TAT TCT TG	
*Leu. citreum*	CI_F	GGT GCA TTG CAC TCG TCA TA	101
	CI_R	AAT GAG AGC GTT GGC TAT CC	
*Leu. fallax*	FA_F	TGT CGC TGA AGG TGG CTA CT	126
	FA_R	GCA CCG CCA TTA TAA GAA ATG AC	
*Leu. gasicomitatum*	GA_F	GAA CCA CCT TTC GAC CAA TTA	103
	GA_R	CAT ACA TTG CGC GAA GTA GC	
*Leu. gelidum*	GE_F	CCG AAA ATA TGA GCG CTT AC	121
	GE_R	GAA TCC ATA CCT GCC TGA AC	
*Leu. holzapfelii*	HO_F	AAC GAC CTA TCG CAC GGA TG	100
	HO_R	AGC GGC GTC AAA GTA GTA CC	
*Leu. inhae*	IN_F	TGG CAC TTG AAC CAG CAT TG	123
	IN_R	CCG TTA CGC CTT CGT TGA TA	
*Leu. kimchii*	KI_F	GGA AAA CTT GCC TCC TCA TTC A	190
	KI_R	GGC GCC TGT GTA TGT ACC AGA T	
*Leu. lactis*	LA_F	CAC TTA ATC GCG CAG AAC AC	102
	LA_R	CCG GCG TTG TGC CTA AGT CA	
*Leu. mesenteroides*	ME_F	CGG TCA ACC AAT ACT TAC CAA GA	211
	ME_R	ATT GAA TTA CTC GCG CTC TG	
*Leu. pseudomesenteroides*	PS_F	AGT GGT GTG GCA GCA GGT AA	171
	PS_R	ACG GCA GCA GTC AAT TCC TT	
*Leu. suionicum*	SU_F	TGA ACA CAA CGG TCA GTA CG	128
	SU_R	CCT GCC ACA ATG GCT CTA GT	

### Specificity and sensitivity of primer pairs for real-time PCR

**Figure 3:**
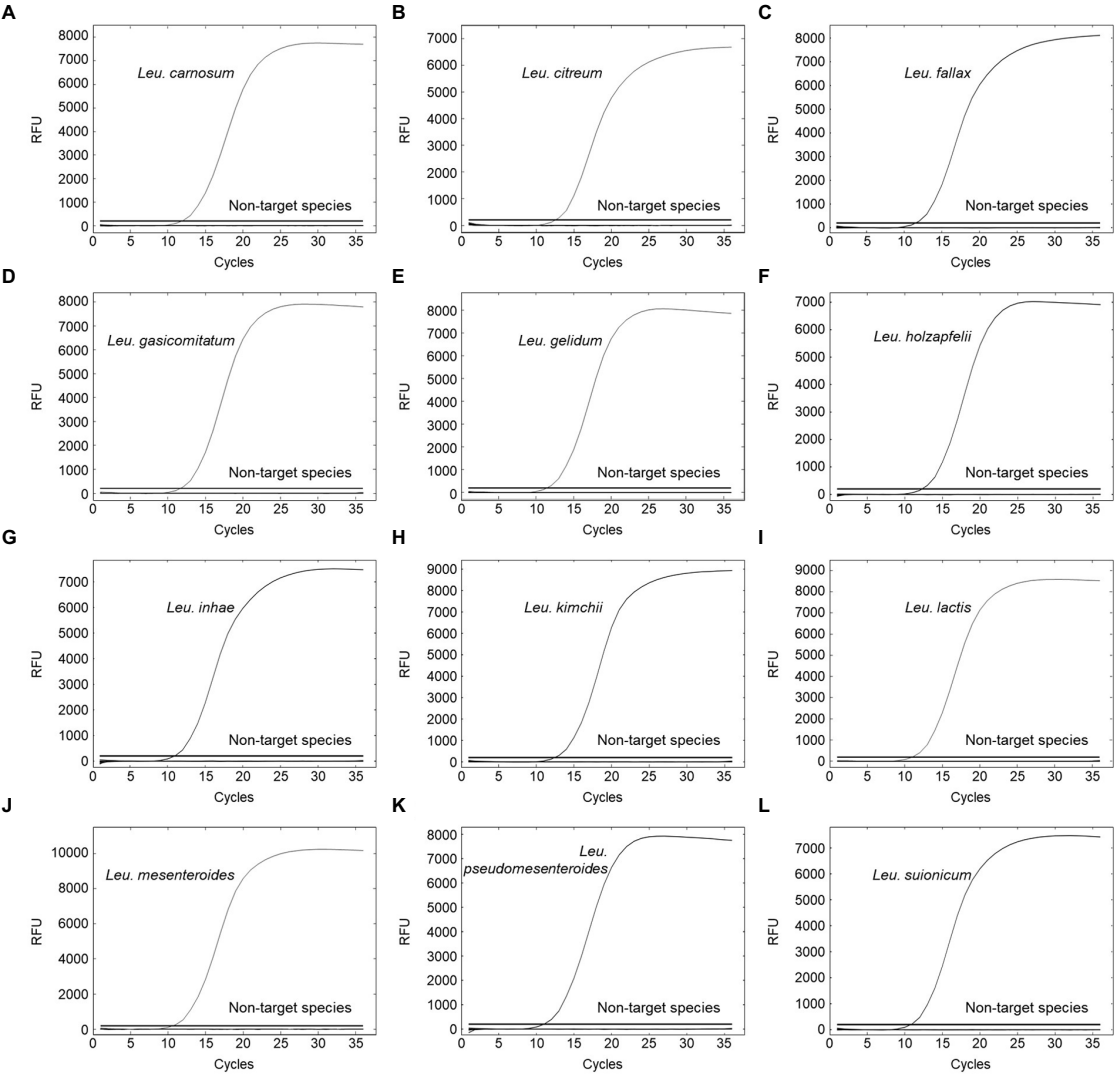
Specificity evaluation for **(A)** *Leu*. *carnosum* primer pair, **(B)** *Leu*. *citreum* primer pair, **(C)** *Leu*. *fallax* primer pair, **(D)** *Leu*. *gasicomitatum* primer pair, **(E)** *Leu*. *gelidum* primer pair, **(F)** *Leu*. *holzapfelii* primer pair, **(G)** *Leu*. *inhae* primer pair, **(H)** *Leu*. *kimchii* primer pair, **(I)** *Leu*. *lactis* primer pair, **(J)** *Leu*. *mesenteroides* primer pair, **(K)** *Leu*. *pseudomesenteroides* primer pair, and **(L)** *Leu*. *suionicum* primer pair. Test samples comprised 23 target strains of *Leuconostoc* species and 124 non-target strains.

**Figure 4:**
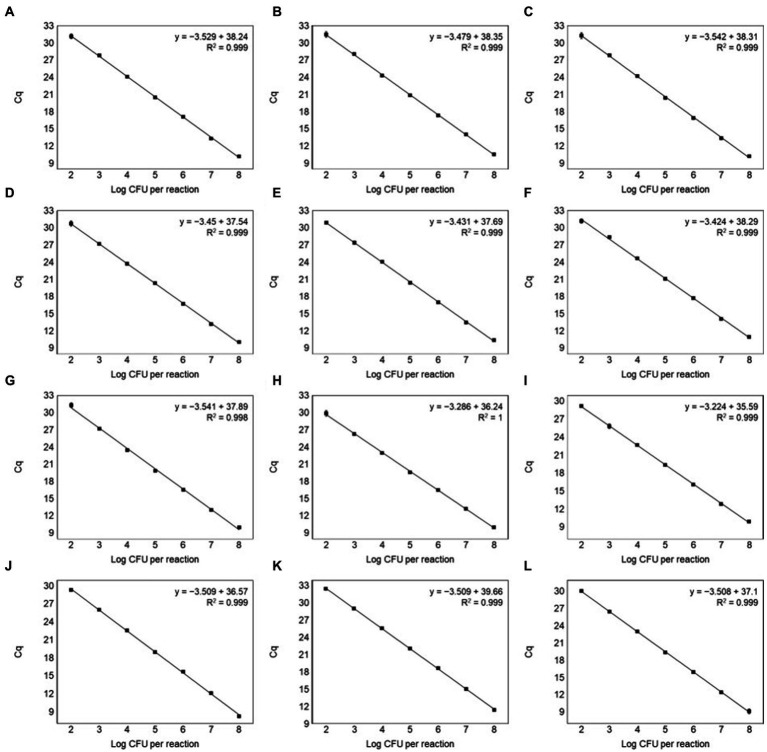
Standard curves by plotting Ct values against the log CFU/ml for **(A)** *Leu*. *carnosum*, **(B)** *Leu*. *citreum*, **(C)** *Leu*. *fallax*, **(D)** *Leu*. *gasicomitatum*, **(E)** *Leu*. *gelidum*, **(F)** *Leu*. *holzapfelii*, **(G)** *Leu*. *inhae*, **(H)** *Leu*. *kimchii*, **(I)** *Leu*. *lactis*, **(J)** *Leu*. *mesenteroides*, **(K)** *Leu*. *pseudomesenteroides*, and **(L)** *Leu*. *suionicum* in *inhae* pure culture. All samples were tested in triplicate.

The specificity of primer pairs was tested using various reference strains available for this study. All target strains produced amplification curves for the corresponding primer pairs. Contrastingly, non-target strains did not produce any amplicons, indicating 100% specificity with no cross-reactivity (Figure 3). This validated the high specificity of the designed primer pairs. The Ct values of the amplification plot ranged from 9.86 to 12.5 (Supplementary Table S6). To calculate the efficiency of primer pairs, we generated standard curves using different concentrations of genomic DNA (10^8^ to 10^1^ CFU/ml) from 12 *Leuconostoc* species. Real-time PCR can detect up to 10^2^ CFU/ml for all target species (Figure 4). Previous reports showed similar or lower sensitivities than our study for other lactic acid bacterial species, such as *Lacticaseibacillus paracasei* (10^2^ CFU/ml) and *Lactobacillus* species (10^3^ CFU/ml) (Jomehzadeh et al., 2020; Hu et al., 2021). To generate valid primer pairs, the slope and correlation coefficient (*R*^2^) for the standard curve should be −3.1 to −3.6 and ≥ 0.98, respectively (Broeders et al., 2014). The slops of the linear regression curves for the 12 *Leuconostoc* species ranged between −3.431 and −3.589, and the amplification efficiencies ranged from 90 to 95.6%, with an *R*^2^ value of ≥0.998. These results indicate that our real-time PCR method using species-specific primer pairs has high detection efficiency.

### Evaluation of the sensitivity in food matrix by real-time PCR

To evaluate the suitability of real-time PCR, genomic DNA was extracted from pork, lettuce, and milk samples artificially contaminated with different concentrations of 12 *Leuconostoc* species. The sensitivity of the target genes in the artificially contaminated food samples was 10^2^ CFU/g, equivalent to pure culture (Supplementary Table S7). Standard curves showed good linear correlations, with *R*^2^ values of ≥0.991 in all the samples (Table 3). The real-time PCR efficiencies ranged between 90.9 and 109.9%.

**Table 3:** The equation and coefficient of correlation values of standard curves in spiked pork, lettuce, and milk samples.

Species	Pork		Lettuce		Milk	
	Equation of standard curve	*R* ^2^	Equation of standard curve	*R* ^2^	Equation of standard curve	*R* ^2^
*Leu. carnosum*	y = −3.445x + 40.87	0.997	y = −3.498x + 40.82	0.999	y = −3.514x + 41	1
*Leu. citreum*	y = −3.235x + 41.44	0.991	y = −3.371x + 42.02	0.995	y = −3.443x + 38.93	1
*Leu. fallax*	y = −3.268x + 38.76	0.997	y = −3.344x + 39.01	0.998	y = −3.21x + 38.38	0.998
*Leu. gasicomotatum*	y = −3.123x + 40	0.993	y = −3.43x + 41.02	0.998	y = −3.44x + 40.91	0.999
*Leu. gelidum*	y = −3.185x + 40.28	0.992	y = −3.377x + 40.38	0.999	y = −3.43x + 39.65	0.997
*Leu. holzapfelii*	y = −3.226x + 41.46	0.994	y = −3.317x + 40.39	0.999	y = −3.422x + 41.47	0.998
*Leu. inhae*	y = −3.562x + 41.4	0.998	y = −3.4x + 41.19	0.999	y = −3.221x + 40.93	0.999
*Leu. kimchii*	y = −3.264x + 39.91	0.996	y = −3.471x + 40.65	0.999	y = −3.47x + 39.79	0.997
*Leu. lactis*	y = −3.214x + 37.91	0.999	y = −3.105x + 37.54	0.998	y = −3.302x + 38.94	0.998
*Leu. mesenteroides*	y = −3.477x + 38.91	0.999	y = −3.307x + 37.02	0.999	y = −3.17x + 36.33	0.999
*Leu. pseudomesenteroides*	y = −3.354x + 38.41	0.999	y = −3.356x + 38.28	0.999	y = −3.378x + 38.52	0.998
*Leu. suionicum*	y = −3.263x + 38.02	0.998	y = −3.327x + 38.44	0.999	y = −3.238x + 37.96	0.998

The sensitivity obtained for the food samples artificially inoculated with *Leuconostoc* species is greater than those reported for other bacterial species in various food samples, such as lactic acid bacteria from cold-smoked salmon (10^3^ CFU/g), *Pseudomonas aeruginosa* in tomato (10^3^ CFU/g), and *Lactobacillus kefiri* in kefir milk (10^3^ CFU/g) (Kim et al., 2016; Jérôme et al., 2022; Wang et al., 2022). Contrastingly, we obtained sensitivity similar to that reported for the detection of *Weissella viridescens* in vacuum-packaged morcilla (10^2^ CFU/ml) (Martins et al., 2020).

As meat, lettuce, and milk are complex foods containing starch, fat, and proteins, the possibility of finding contaminants in the extracted genomic DNA is higher. These components might affect the sensitivity and efficiency of the PCR reactions, as described in previous reports. Our real-time PCR method showed good linearity with regard to the standard curves for 12 *Leuconostoc* species inoculated into the three food types, suggesting that it is not affected by inhibitors (Zhou et al., 2022). This result indicates that the screened targets have good sensitivity and anti-interference ability for real-time PCR to identify *Leuconostoc* species rapidly and accurately in artificially spiked food samples.

## Conclusion

To our knowledge, this is the first study demonstrating a novel real-time PCR-based method for identifying and discriminating between the major *Leuconostoc* species found in foods. Our real-time PCR method utilized newly discovered marker genes highly specific for identifying 12 *Leuconostoc* species, which displayed high specificity and good consistency for *Leuconostoc* species detection. Our qPCR method enabled rapid, specific, and sensitive *Leuconostoc* identification. It might be used as an alternative molecular method to identify these *Leuconostoc* species in food samples and possibly identify novel food-based *Leuconostoc* strains in the future.

## Data availability statement

The datasets presented in this study can be found in online repositories. The names of the repository/repositories and accession number(s) can be found in the article/Supplementary material.

## Author contributions

EK and H-YK contributed to conception and design of this study. EK and S-MY performed pangenome analysis and unique gene extraction. I-SK and S-MY performed the experimental work. S-YL analyzed average nucleotide identity. H-YK supervised the work and reviewed and edited the manuscript. EK prepared a draft manuscript. All authors contributed to manuscript revision, read, and approved the submitted version.

## Funding

This work was carried out with the support of “Cooperative Research Program for Agriculture Science and Technology Development (Project No. PJ01662001)” Rural Development Administration, Republic of Korea.

## Conflict of interest

The authors declare that the research was conducted in the absence of any commercial or financial relationships that could be construed as a potential conflict of interest.

## Publisher’s note

All claims expressed in this article are solely those of the authors and do not necessarily represent those of their affiliated organizations, or those of the publisher, the editors and the reviewers. Any product that may be evaluated in this article, or claim that may be made by its manufacturer, is not guaranteed or endorsed by the publisher.

## Footnotes

^1^ https://lpsn.dsmz.de/genus/leuconostoc

^2^ http://insilico.ehu.es/PCR/
